# Severe seizures in pigs naturally infected with *Taenia solium* in Tanzania

**DOI:** 10.1016/j.vetpar.2016.02.025

**Published:** 2016-04-15

**Authors:** Chiara Trevisan, Ernatus M Mkupasi, Helena A Ngowi, Björn Forkman, Maria V Johansen

**Affiliations:** a Department of Veterinary Disease Biology, University of Copenhagen, Dyrlægeve*j* 100, 1870 Frederiksberg C, Denmark; b Department of Veterinary Medicine and Public Health, Sokoine University of Agriculture, P.O. Box 3021, Morogoro, Tanzania; c Department of Large Animal Sciences, University of Copenhagen, Grønnegårdsve*j* 8, 1870 Frederiksberg C, Denmark

**Keywords:** Seizures, *Taenia solium* cysticerci, Neurocysticercosis, Pigs, Epilepsy, Behaviour, Animal welfare, Tanzania

## Abstract

Neurocysticercosis (NCC) caused by *Taenia solium* is a serious neurological disease. In humans neurological symptoms have been thoroughly studied and documented, however, there is limited information on clinical signs in pigs infected with *T. solium* cysticerci. Among the scientific community, it is in fact believed that pigs with NCC rarely show neurological signs. The aim of this study was to describe clinical manifestations associated with NCC in pigs and correlate the manifestations to the number and distribution of cysticerci in brains of naturally infected pigs in Tanzania. Sixteen infected and 15 non-infected control pigs were observed for 14 days during daylight hours, and subsequently videotaped for another 14 consecutive days using close circuit television cameras. All occurrences of abnormal behaviour (trembling, twitching, mouth and ear paralysis, ataxia, dribbling, salivating, eye blinking, walking in circles) were recorded. At the end of the recording period, pigs were slaughtered and their brains dissected, cysticerci counted and locations noted. During the recording period, two infected pigs were observed having seizures. Some of the observed autonomic signs during a seizure were chewing motions with foamy salivation and ear stiffening. Motor signs included tonic muscle contractions followed by a sudden diminution in all muscle function leading to collapse of the animal. Stereotypic walking in circles was observed on several occasions. At dissection, both pigs had a high number of brain cysticerci (241 and 247 cysticerci). The two pigs with seizures were also older (36 months) compared to the others (18.3 months, ± 8.2 standard deviation). Results of this study have shown that pigs with NCC can develop clinical signs and suffer from seizures like humans with symptomatic NCC. Results of this study could potentially open up a new experimental pathway to explore the aetiology of neurological symptoms in humans with NCC associated epilepsy.

## Highlights

• Pigs with neurocysticercosis can develop clinical signs and suffer from seizures.
• Autonomic signs were chewing motions with foamy salivation and ear stiffening.
• Motor signs included tonic muscle contractions and tremor.
• Stereotypic walking in circles was observed on several occasions.

## Graphical abstract

**Figure:**
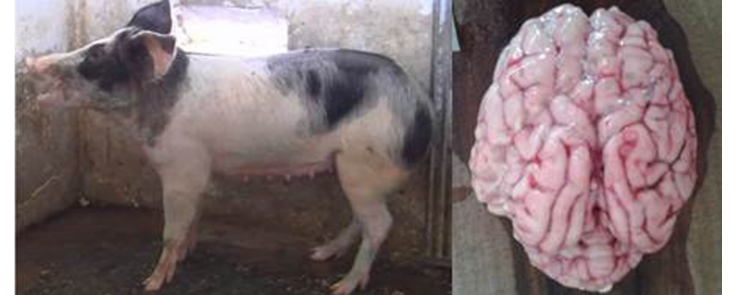

## 1 Introduction

Neurocysticercosis (NCC) caused by the larval stage of the pig tapeworm *Taenia solium* is a serious neurological disease (White, 1997). In humans, epilepsy, headache and impaired vision are common clinical presentations of NCC and leading causes of morbidity. Dementia, learning difficulties and changes in cognition are often secondary sequelae of humans with NCC (Garcia et al., 2003). In endemic areas NCC is estimated to be responsible for one third of all late onset epilepsy cases (Ndimubanzi et al., 2010) and causes substantial health and economic burdens in the affected populations (Carabin et al., 2006, Praet et al., 2009, Torgerson and Macpherson, 2011, Trevisan et al., 2016).

Whilst in humans these neuropathologic and clinical aspects have been well studied and documented, there is limited information on the symptomatology the parasite causes in pigs. A number of researchers have previously reported the rarity of signs developed in association with porcine cysticercosis and a limited number of studies exploring the latter (Garcia et al., 2003, Saenz et al., 2008). However, in a study conducted in India, eye blinking, tearing and excessive salivation were reported as clinical signs suggestive of porcine NCC. To confirm presence of cysticerci in the brain and correlate the signs to NCC, the pigs underwent magnetic resonance imaging (MRI) examination (Prasad et al., 2006). Since the animals included in the study of Prasad et al. (2006) were chosen based on the signs observed by animal caretakers and as the pigs had to be sacrificed for gross pathology and histopathology examination at the end of the MRI scans, the signs could not be reconfirmed. Furthermore, as the study design did not include control pigs, it is not known if the reported signs occurred due to the health conditions of the animals or their environment during the study. Another study by Mkupasi et al. (2014) reported clinical manifestations such as dullness, sluggishness, somnolence, apathy and loss of consciousness in pigs naturally infected with *T. solium* cysticerci. The observed clinical manifestations were only considered as suggestive of NCC because no control pigs were available in that study.

In pigs, seizures have been observed to be caused by salt poisoning, dehydration or pseudo rabies (Gelberg, 2010), however, the zoonotic parasite *T. solium* has never been reported as an agent causing seizures in this species.

The aim of the present study was to describe possible clinical manifestations associated with NCC and correlate the manifestations to the number, location and distribution of cysticerci in brains of naturally infected pigs in Tanzania.

## 2 Materials and methods

### 2.1 Animals

The study was carried out at the experimental animal facilities at Sokoine University of Agriculture (SUA), Morogoro, Tanzania. Infected pigs were purchased from farmers in the rural area of Kongwa district, Dodoma region, an area where *T. solium* is known to be highly prevalent (Mkupasi et al., 2013). To ascertain presence of infection, the pigs were diagnosed by tongue examination (Dorny et al., 2004). Pigs with more than three cysticerci under the tongue were included in the study. As the sensitivity of the diagnostic method is low, non-infected pigs were purchased from smallholder farmers in villages of Morogoro rural district, Morogoro region, where the prevalence of porcine cysticercosis is known to be low (Ngowi, personal communication).

Pigs not sexually mature (maturity determined by age (younger than 6 months) and size (lower than 50 cm in height) or in poor condition (under two thirds of the average weight of 40 kg for a healthy adult pig and visibly ill (covered with ectoparasites and/or with injuries)) were excluded. Infected pigs were purchased first and non-infected pigs were matched with infected ones a posteriori by applying the same exclusion criteria. On arrival of the pigs at SUA, all animals were checked for hard ticks and lice. To eliminate possible confounders (endoparasites and ectoparasites) pigs were treated twice, at an interval of two weeks, with a subcutaneous injection of 0.3 mg/kg of ivermectin (ivermectin 1.0%) (Barragry, 1987). Ivermectin was considered safe, as no adverse effects were observed in a study on ivermectin in pigs with cysticercosis (Mkupasi et al., 2013).

### 2.2 Study design

An observational study was carried out on 16 pigs naturally infected with *T. solium* and 15 non-infected pigs. A parallel group design was formed. As the disease cannot spread between pigs, three to four infected pigs and their respective controls were randomly chosen and housed together. All pigs were housed under equal conditions in pens (4 × 3 m) with a cement floor and walls. The pens were cleaned every day and the pigs were fed twice a day with commercial dry pig feed. Water was provided *ad libitum* and after morning feeding the animals were provided with forage (*Leucaena leucocephala* or *Amaranthus spinosus*). The mean room temperature of the stable was of 25 °C. Light was present from 6:00 am until 7:00 pm.

Pigs were kept at the experimental animal facilities for one month. During the first two weeks of acclimatisation, the pigs were observed during the day (7:30 am until 6:00 pm). Abnormal occurrences such as: trembling, twitching, uncontrolled movements of rostrum, mouth rigidity and ear paralysis, eye blinking, dribbling, salivating, body stiffening, ataxia, tonic/clonic contractions, panting, collapse of the animal and stereotypic walking in circles were recorded when observed.

After two weeks of acclimatisation, the pigs were continuously videotaped for 14 consecutive days using close circuit television cameras (Velleman^®−^ CCTVPROM16). One camera was mounted above each pen in a central position, permitting a top down view of the whole pen. Each pig was colour-marked using coloured stock markers on its back and on its sides to allow individual identification on the video recordings. The videos of the animals where abnormal occurrences were observed during the acclimatisation period were scanned for abnormal behaviour using fast forward video visualization.

At the end of the video recording period, all 31 pigs (infected and non-infected) were slaughtered by a local butcher. After bleeding, the carcasses of the pigs were transported to the pathology laboratory of the Department of Veterinary Pathology, Faculty of Veterinary Medicine at SUA. There the animals were decapitated at the atlanto-occipital joint (Agerholm, 2011). To remove the brain from the cranium the procedure described by Agerholm (2011) was adopted.

To evaluate the distribution of the cysticerci, the cerebral part of the brain was divided into its left and right hemisphere. Thereafter each hemisphere was divided into frontal, temporal, parietal and occipital lobe and cerebellum using sulci as landmarks. Each lobe was carefully sliced to examine and enumerate cysticerci. The cysticerci were evaluated to determine whether they were located in the extra-parenchymal part (parts not completely surrounded by brain tissue) i.e. subarachnoid dorsal and subarachnoid base or in the parenchymal part of the frontal, temporal, parietal and occipital lobe and cerebellum. Cysticerci that could not be located were counted as cysticerci found on the cutting board. Furthermore, the cysticercus developmental stage (vesicular, colloidal or calcified) was recorded (Fleury et al., 2004).

### 2.3 Seizure classification

Seizures were classified according to a modified version of the International League Against Epilepsy (ILAE) seizure system developed for dogs (Licht et al., 2002). The seizure classification summary (Table S1) used is available in Supplementary material. Seizures were divided into four categories: Partial, complex partial, partial with secondary generalization and generalized (Licht et al., 2002).

### 2.4 Data analysis

Data were entered in Microsoft Excel 2010 and analysed using the statistical software R (R Core Team, 2014). Descriptive statistics were performed to compute means and proportions of cysticerci in different parts of the brain. Multiple logistic regression was adopted to assess the effect of age, total number and location of cysticerci on presence of seizures. P-values lower than 0.05 were considered statistically significant.

### 2.5 Research ethics

Practices employed in the study were approved by SUA, Morogoro, Tanzania (Ref. no. RPGS/R/AS/42/2014) and in accordance with the national guidelines of ethics for health research and to the Animal welfare act (2008) (Mashalla et al., 2009, The United Republic of Tanzania, 2008).

## 3 Results

### 3.1 Seizures

During the study period two infected pigs were observed having seizures. Seizures occurred recurrently in both pigs. The average seizure duration was 20:51 minutes (Standard Deviation (SD) 14:20 minutes). Video clip 1–7 (available in the electronic version) show the abnormal behaviours observed during a seizure.

#### 3.1.1 Description of seizures in pig 1

Observed autonomic signs were chewing motions with foamy salivation and dribbling, ear rigidity, eye blinking and panting. Motor signs included tremor, ataxia and tonic/clonic muscle contractions followed by a sudden diminution of all muscle function leading to collapse (Video clip 1—available in the electronic version). Stereotypic walking in circles was observed in several occasions after the occurrence of the autonomic and motor signs (Video clip 2—available in the electronic version). Vomiting also occurred during a seizure (Video clip 3—available in the electronic version). During the post-ictal phase the pig gave the impression of being disoriented as bumping into walls and stumbling over the feeding and water trough was observed (Video clip 4—available in the electronic version). According to the modified version of the ILAE seizure systems, seizures in pig 1 were classified as partial with secondary generalization and generalized.

#### 3.1.2 Description of seizures pig 2

Observed autonomic signs were uncontrolled movements of mouth and rostrum, dribbling, eyes widening, blinking and ear paralysis. Others signs were brief respiratory arrests, mouth paralysis with continuous swallowing movements of throat and tongue. Also chewing and salivating were common. Observed motor signs included tonic muscular contractions and/or clonic movements like trembling of certain body parts or regions (e.g. head or whole body). Body stiffness and ataxia followed (Video clip 5—available in the electronic version). Other observed motor signs were unwanted repeated lifting of one limb and unbalanced walking. Stereotypic walking in circles was also observed in several occasions during a seizure (Video clip 6 and 7—available in the electronic version). Consciousness seemed to be preserved during partial seizures, while impaired during secondary generalization. According to the modified version of the ILAE seizure systems, seizures in pig 2 were classified as partial seizures, complex partial and partial with secondary generalization.

### 3.2 Cysticercus distribution and location

Brain cysticerci were found in all 16 pigs classified as *T. solium* infected by tongue examination. No cysticerci were found in any of the 15 control pigs. Cysticerci found in the brains were vesicular and colloidal. Calcified cysticerci were not detected in any of the brains. The number of cysticerci varied from 4 to 418 cysticerci, with a median of 67.5 cysticerci and a mean of 106.7 ± 28.8 standard error of the mean (Table 1). Table 2 describes the distribution and localisation of cysticerci found in the 16 infected pigs. The mean age of the pigs was 18.3 months ± 8.2 standard deviation (Table 1). Although only two pigs were observed having seizures, multiple logistic regression analysis found this to be significantly related to age (p < 0.001) but not to total number, distribution and localisation of cysticerci in the brain.

## 4 Discussion

This is the first study that has monitored a group of non-infected pigs and pigs naturally infected with *T. solium* in a controlled environment continuously for one month. Results of this study have shown that pigs with NCC can develop clinical signs and suffer from seizures like humans with NCC associated epilepsy.

In this study, seizures could be detected as the animals were extensively monitored many days in a row. In sub-Saharan Africa pigs are usually left to roam freely and therefore neither controlled nor observed by their owners. Furthermore, pig farmers might not be aware of seizures in pigs or if occurring they might relate it to “possession of bad spirits” as in sub-Saharan Africa, epilepsy is believed to be contagious and caused by witchcraft or evil spirits (Carod and Vazquez-Cabrera, 1998, Winkler et al., 2010). People with epilepsy are stigmatized; hence, if farmers observed seizures in pigs, they might fear stigmatization and omit to report it to authorities or researchers. This might potentially explain the lack of previous documentation of seizures in pigs by pig farmers.

The occurrence of seizures and their average duration in this study were in line with studies on epilepsy in other animal species, where they were also observed to vary largely (Licht et al., 2002).

The autonomic and motor signs observed during seizure manifestations in the two pigs were consistent with signs observed in seizures of other species (Licht et al., 2002). Two studies had previously reported signs of NCC in pigs (Mkupasi et al., 2014, Prasad et al., 2006). Prasad et al. (2006) reported eye blinking, tearing and excessive salivation to be suggestive signs of NCC. In this study eye blinking and salivation were observed, however not to an extent that these signs could be useful to identify pigs with cysticercosis. Abnormal signs observed in this study were more in line with those of Mkupasi et al. (2014); however dullness, sluggishness and apathy could not be reported, as a clear definition of the terms was not provided by the authors.

In this study, age has shown to be a factor associated with seizures, with older pigs being more prone to express seizures compared to younger ones. This corresponds with knowledge obtained from studies in humans, where seizures were reported to be a frequent manifestation in patients with degenerating cysticerci (Garcia et al., 2014). Moreover the probability of older pigs having degenerating cysticerci is higher compared to younger pigs (de Aluja and Vargas, 1988, Saenz et al., 2008). Vesicular and colloidal cysticerci were found in both pigs with seizures showing on-going immuno-inflammatory reaction in the brain. This is also in line with studies carried out in humans, where patients suffering from NCC had prominent immuno-inflammatory reactions provoking parasite destruction and leading to clinical manifestations (Fleury et al., 2004).

In the present study, no associations were observed between seizures and number, distribution and localisation of cysticerci in the pig brain. Similar results were also obtained in a study on 29 symptomatic NCC patients, where no significant associations were observed between presence of seizures and number and localisation of the cysticerci in the human brain (Prasad et al., 2008).

In this study, both pigs with seizures had a high number of cysticerci, while in humans also a single cysticercus can lead to seizures (Padma et al., 1994).

Results of study carried out on 20 symptomatic NCC patients by Saenz et al. (2008) showed symptoms to be significantly associated with the presence of cysticercus in the subarachnoid base of the human brain. In the two pigs of this study that showed seizures, the latter was not observed. The latter might be explained by the fact that symptomatic subarachnoid NCC in humans takes years to develop symptoms (Fleury et al., 2004).

Finally, Saenz et al. (2008) found clear differences between porcine and human cysticercosis however, our results run contrary to those conclusions. The clinical and biological similarities with human NCC associated epilepsy found in this study together with the functional and anatomical similarities of the pig and human brain make the pig a promising model to further understand the aetiology of epilepsy and the relationship between epilepsy and NCC in both humans and pigs (Sauleau et al., 2009).

Animals were treated with ivermectin twice at the beginning of the study. An inflammatory response around cysts which may lead to seizures cannot be ruled out, however, in a study by Mkupasi et al. (2013) no adverse effects were observed when ivermectin was used in pigs with cysticercosis (Mkupasi et al., 2013). A study on humans, reported that ivermectin may have an effect on human cysticercosis, however the authors concluded that ivermectin did not give rise to any side effects in treated patients (Diazgranados-Sanchez et al., 2008).

## 5 Conclusion

Results of this study have shown that pigs with NCC can develop clinical signs and suffer from seizures like humans with NCC associated epilepsy. Results of this study could potentially open up a new experimental pathway to explore the aetiology of neurological symptoms in humans. However, further studies are warranted to deepen our knowledge on why some pigs with NCC suffer from seizures, while others are asymptomatic. Moreover pigs with NCC and especially those clinically affected should be further studied, as the animal welfare aspect should not be neglected.

## Figures and Tables

**Table 1:** Number of brain cysticerci, pig age in months and presence or absence of seizures in 16 *T. solium* naturally infected pigs.

Pig ID	No of brain cysticerci	Pig age (months)	Seizures
1	247	36	1
2	241	36	1
3	47	18	0
4	418	18	0
5	4	24	0
6	178	12	0
7	4	24	0
8	88	12	0
9	27	12	0
10	21	18	0
11	36	8	0
12	90	12	0
13	10	8	0
14	117	18	0
15	41	18	0
16	136	18	0

**Table 2:** Distribution and localisation of brain cysticerci found in 16 *T*. *solium* naturally infected pigs.

	Right hemisphere		Left hemisphere		Both hemispheres	
	No of cysticerci	%	No of cysticerci	%	No of cysticerci	%
Distribution						
Frontal	309	34.0	281	37.6	590	36.3
Temporal	200	18.0	149	24.3	349	21.5
Parietal	114	19.8	164	13.9	278	17.1
Occipital	179	26.8	222	21.8	401	24.7
Cerebellum	3	0.4	3	0.4	6	0.4
Ventricles	17	1.0	8	2.1	25	1.5
Location						
Subarachnoid dorsal	356	44.2	347	42.4	703	43.3
Subarachnoid base	41	5.1	35	4.3	76	4.7
Parenchyma	408	50.7	437	53.3	845	52.0
Cutting board					58	

*No: number.
