# Supplementary material for: Severe seizures in pigs naturally infected with Taenia solium in Tanzania
Source: Vet Parasitol. 2016 Apr 15;220:67–71. doi: 10.1016/j.vetpar.2016.02.025 (PMC4819911; doi:10.1016/j.vetpar.2016.02.025)
Supplement: Supplementary file 1 [file mmc1.docx]

# Table S1: Seizure classification summary used in dogs (Licht et al. (2002))

| I. Partial seizures |
| --- |
| (A) General criteria. Clinical signs suggest involvement of one part of one cerebral hemisphere. Motor signs are unilateral or asymmetric or involve only limited parts of the body (e.g., head only). May involve unusual behaviours (see Section IB2b). |
| (B) Distinction between SPS and CPS. ^b^ SPS involves preserved consciousness and CPS involves impaired consciousness. Three levels of consciousness are defined. |
| 1. Lost consciousness: Owner's answer to a standard ‘‘responsiveness’’ question indicates that he/she cannot get dog's attention by any method and dog is not navigating environment in any way (e.g. dog not walking, running, or jumping). |
| 1. Impaired consciousness: Dog does not meet both criteria for ‘‘lost’’ consciousness and does meet one or more of the following criteria:   (a) Owner's answer to ‘‘responsiveness’’ question indicates altered attention (i.e., either that he/she cannot get dog's attention or that he/she can get, but not keep, dog's attention).  (b) Dog's behaviour is judged by coder to be ‘‘out of context’’ (e.g. running frantically as if being chased, cowering or hiding for no reason, aggression in otherwise nonaggressive dog).  (c) Owner explicitly says dog was disoriented.  (d) Automatisms are reported.  (e) Postictal disorientation is reported.   1. Preserved consciousness: Owner's answer to ‘‘responsiveness’’ question indicates normal attention and dog does not meet criterion for impaired consciousness. |
| 1. s |
| II. Generalized seizures |
| (A) General criteria. First clinical signs suggest involvement of both cerebral hemispheres. Movements are bilateral and largely symmetrical. If seizure lasts 30 s or longer, consciousness must be ‘‘lost’’ at some point during the seizure to be classified as generalized. |
| (B) Specific types. Unless noted otherwise, clinical signs of each type are defined largely the same as in the ILAE system.   1. Tonic. 2. Clonic. 3. Tonic–clonic: includes generalized seizures involving both tonic and clonic components, regardless of the ordering of components (e.g. clonic–tonic–clonic) or whether the tonic and clonic components occur simultaneously. 4. Myoclonic. 5. Typical absence. 6. Atonic. |
| III. Other terms and classification rules |
| (A) Automatisms. Seizure-related behaviours coded as automatisms include chewing or swallowing movements, lip smacking, licking mouth, licking (grooming) or scratching body, rubbing face or body part, coordinated paddling of four legs. Changing positions or circling is coded as automatism if done more than twice. |
| (B) Cluster. Two seizures that occur less than 24 h apart. |
| (C) Prodrome. When seizure is preceded by 1 h or more of attention seeking, irritability, or anxious behaviour without impairment of consciousness and without motor signs. If this lasts less than one hour, it is classified as a simple partial onset. |
| (D) Progression of a seizure. When a partial seizure progresses to a generalized seizure, it is coded as a partial seizure (SPS or CPS) that secondarily generalized. The progression of an SPS to CPS was difficult to identify reliably. Thus, if this kind of episode occurred, it was coded as CPS. |

b SPS, simple partial seizure(s); CPS, complex partial seizure(s)
