# Supplementary figures and images for: Severe seizures in pigs naturally infected with Taenia solium in Tanzania
Source: Vet Parasitol. 2016 Apr 15;220:67–71. doi: 10.1016/j.vetpar.2016.02.025 (PMC4819911; doi:10.1016/j.vetpar.2016.02.025)

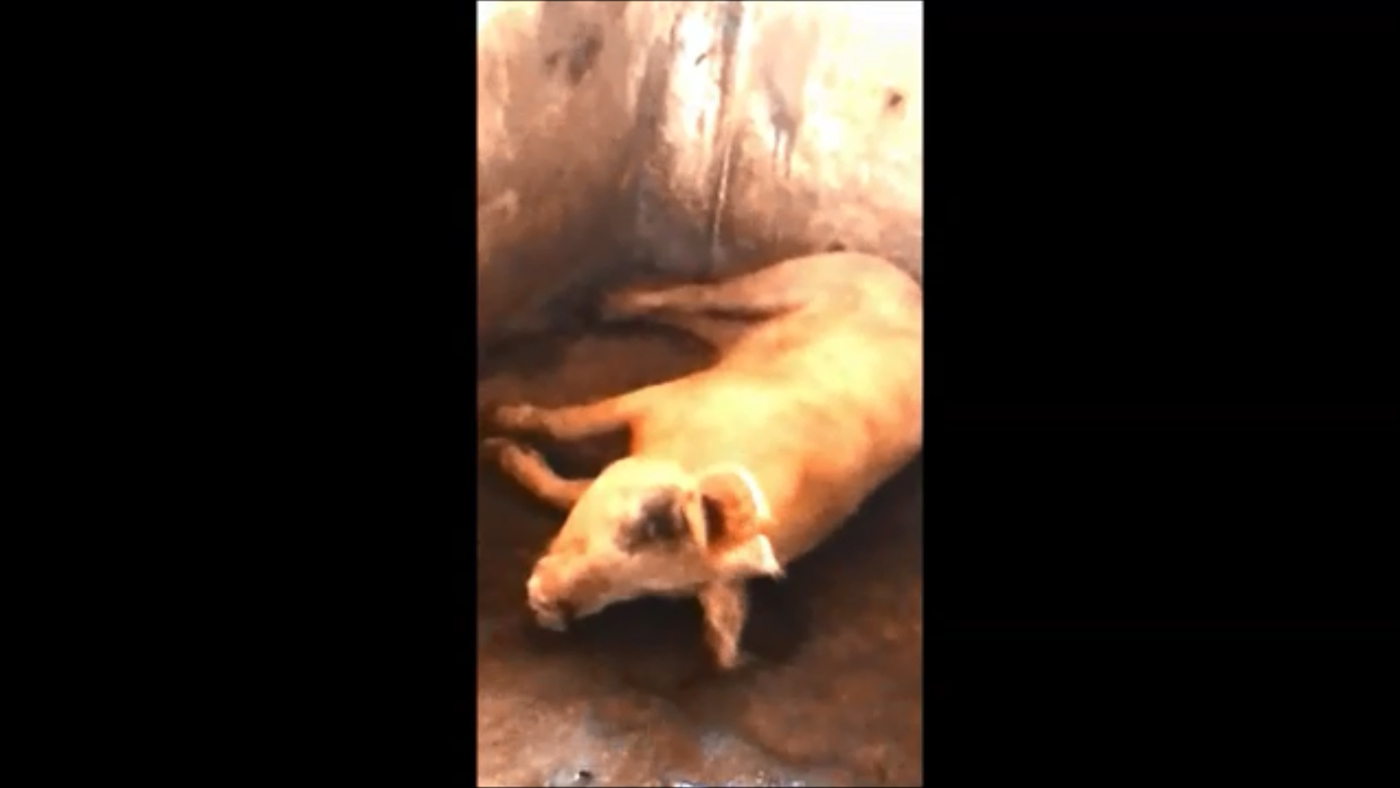

Supplement: Supplementary file 2 [file mmc2.jpg]

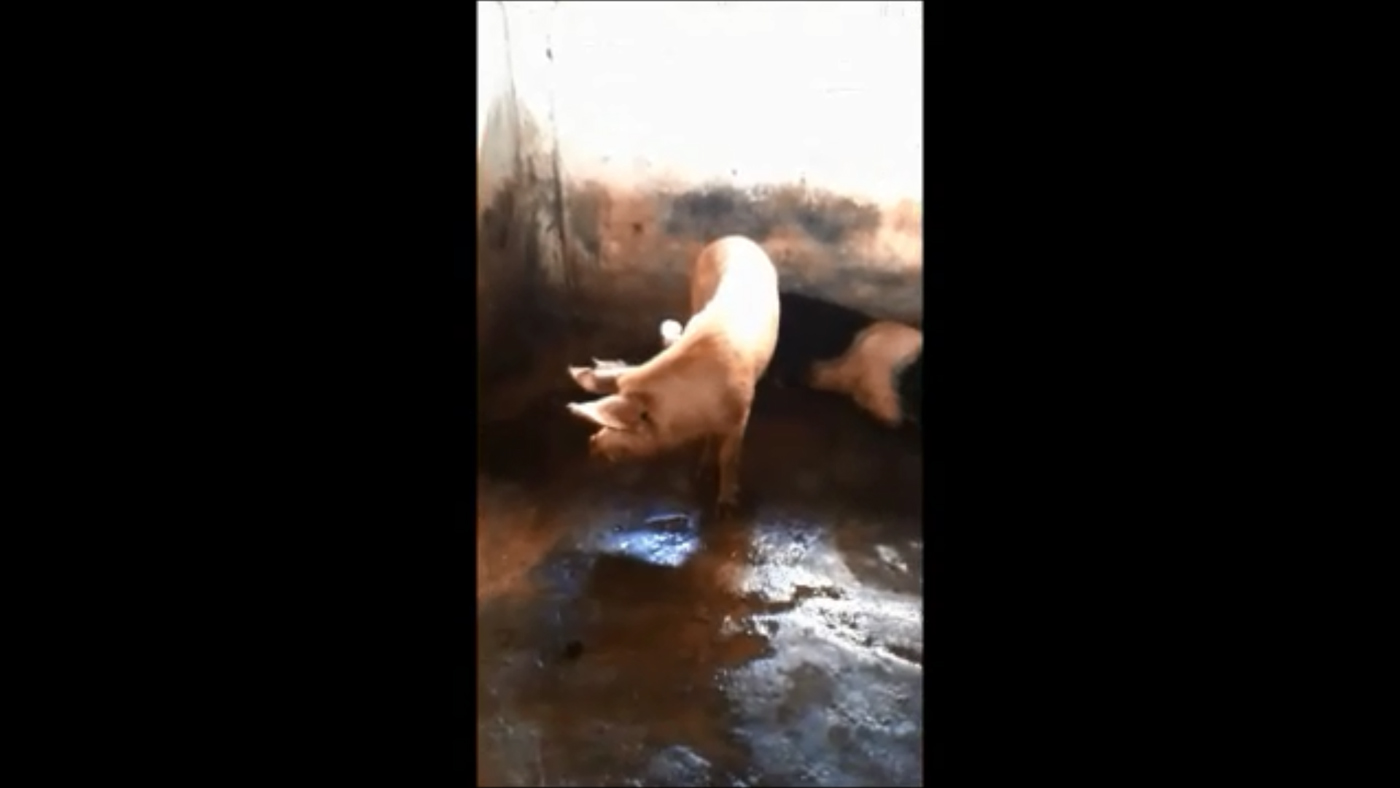

Supplement: Supplementary file 3 [file mmc3.jpg]

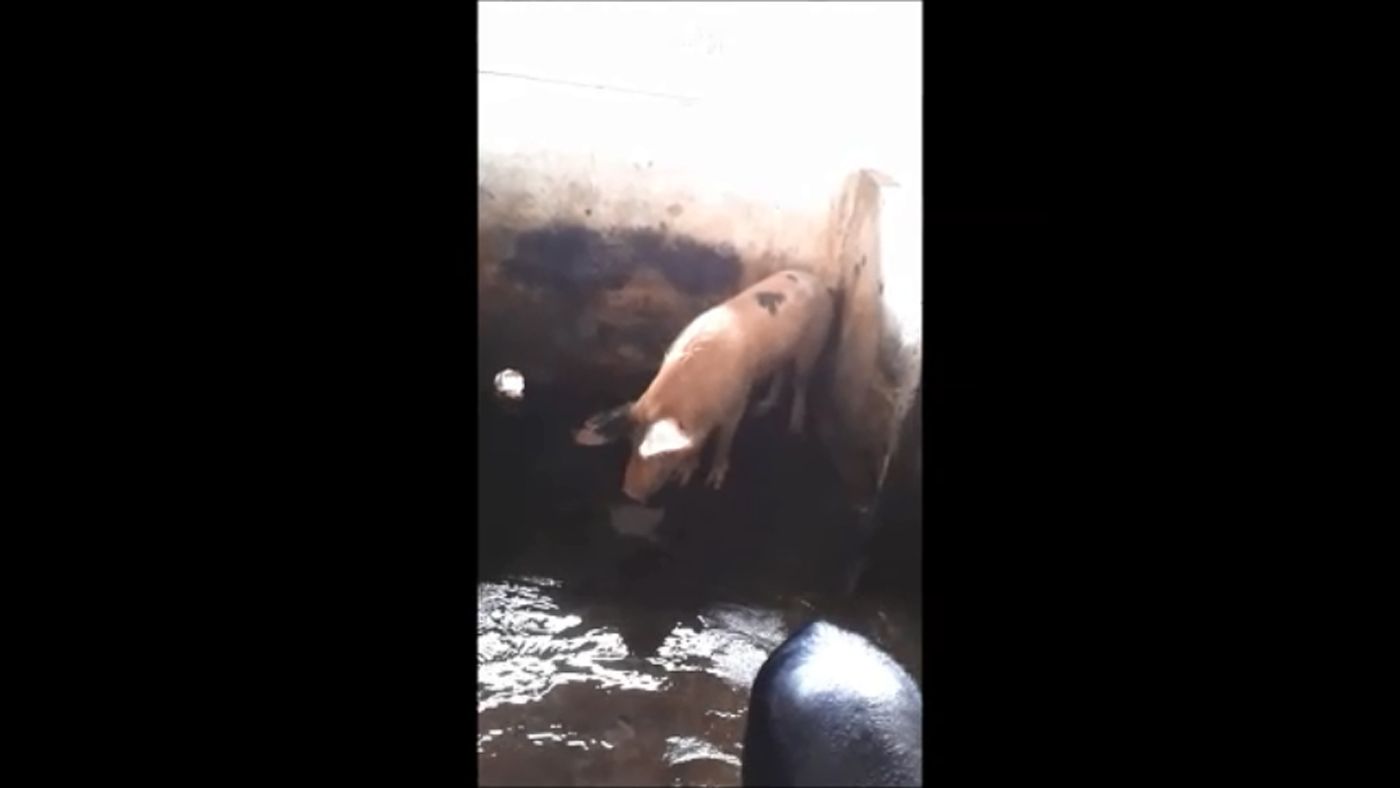

Supplement: Supplementary file 4 [file mmc4.jpg]

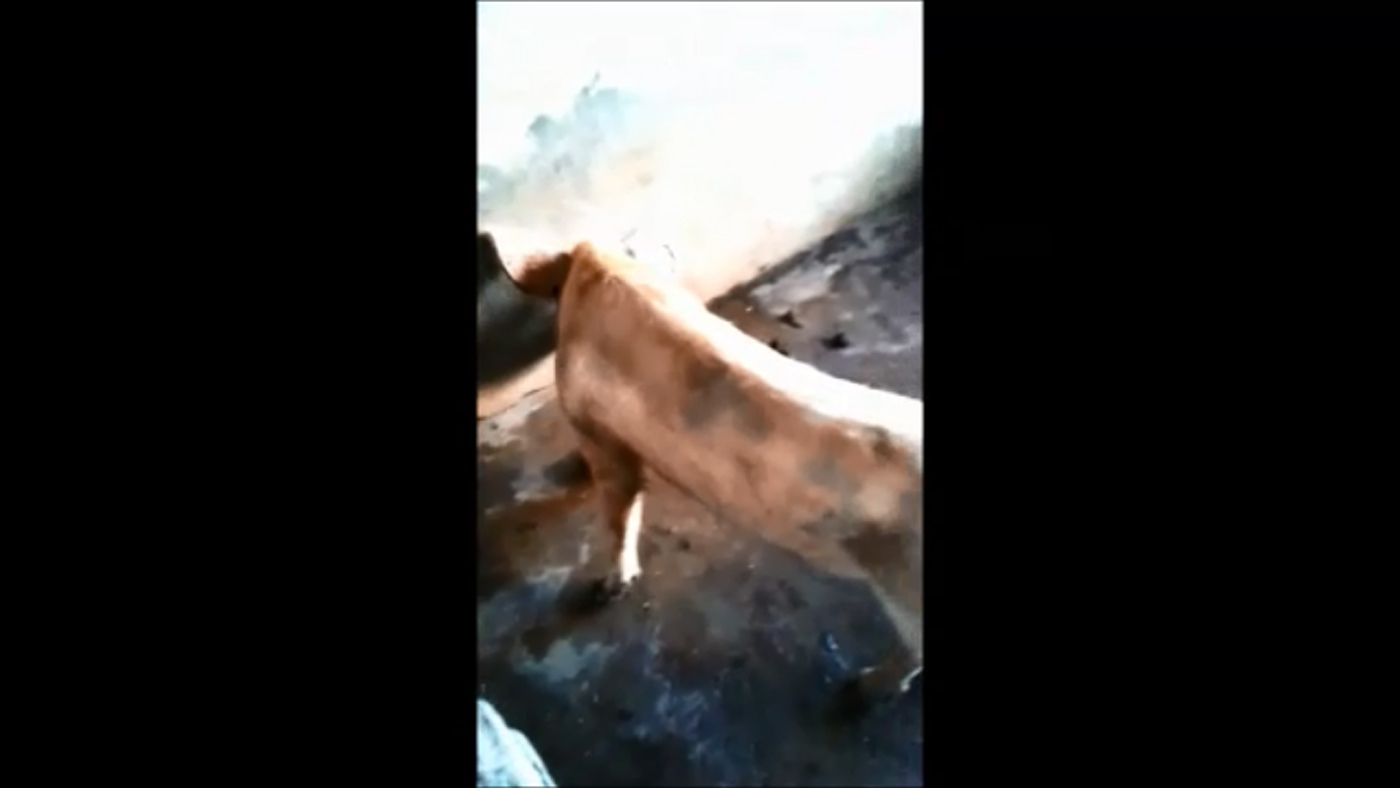

Supplement: Supplementary file 5 [file mmc5.jpg]

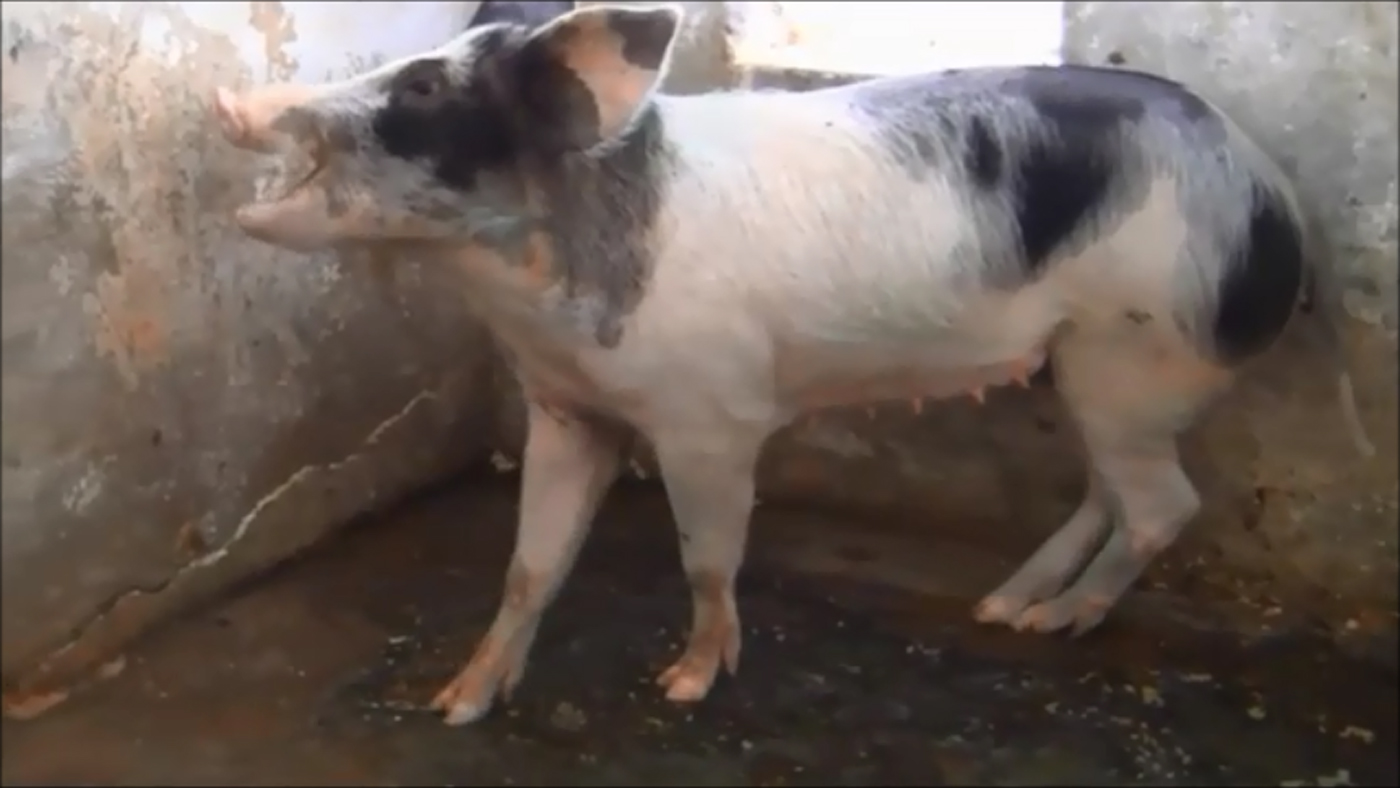

Supplement: Supplementary file 6 [file mmc6.jpg]

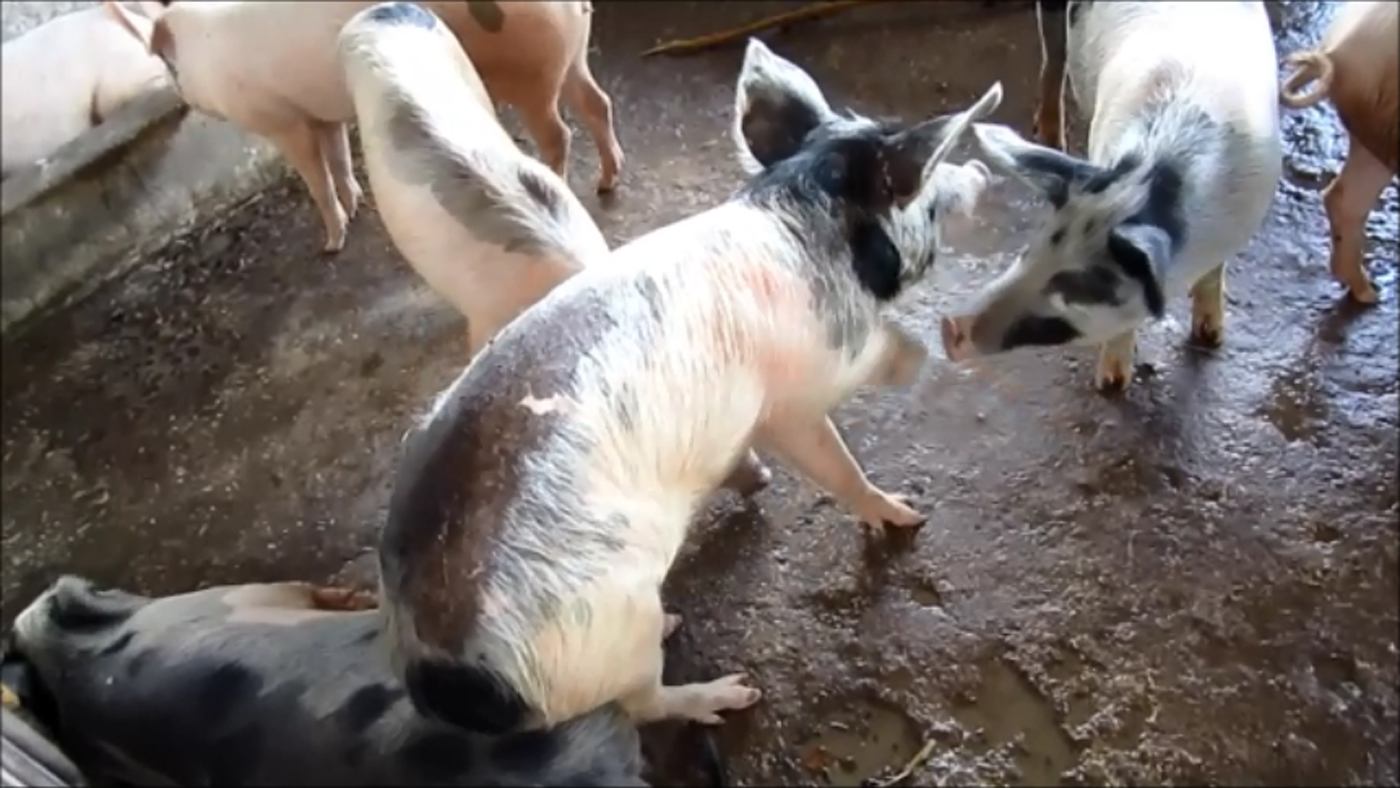

Supplement: Supplementary file 7 [file mmc7.jpg]

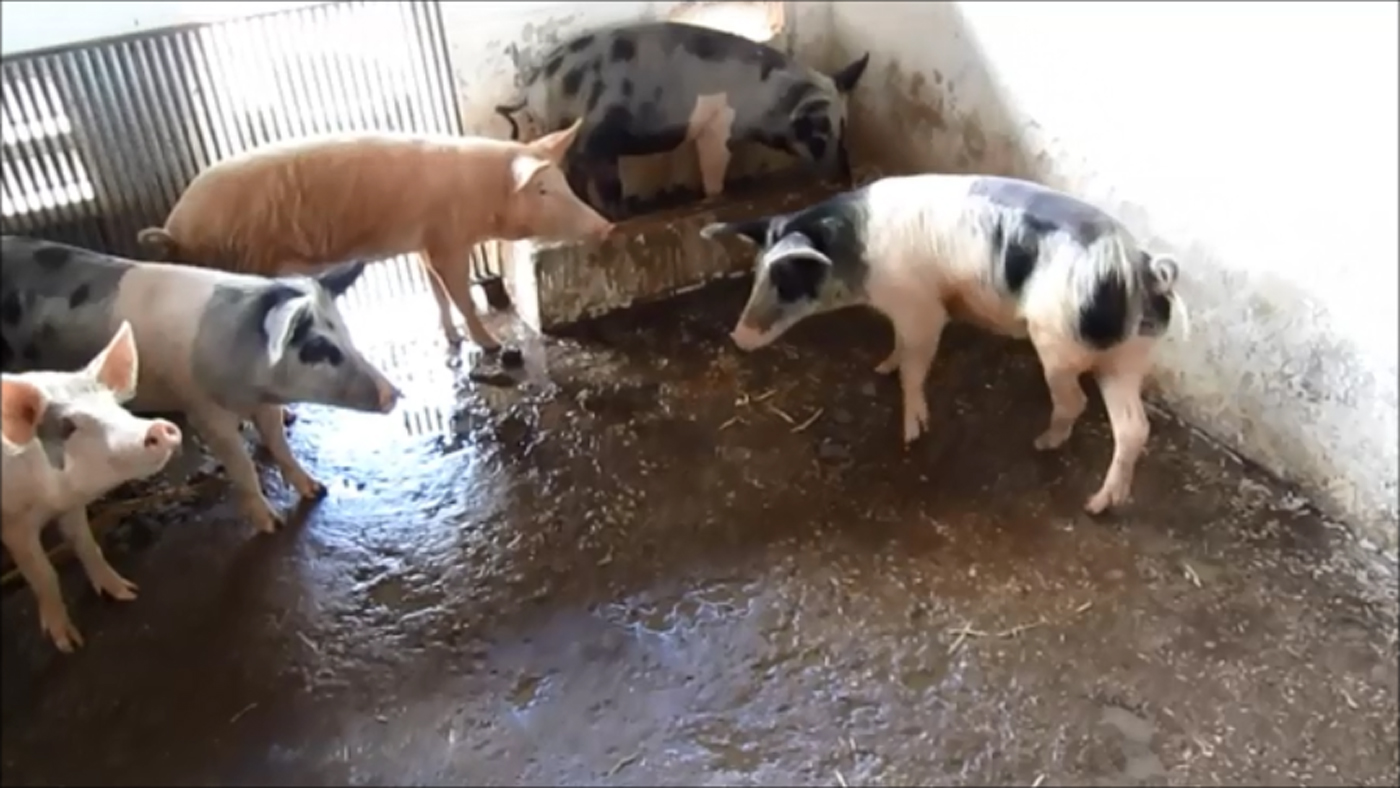

Supplement: Supplementary file 8 [file mmc8.jpg]
